# Seasonal and Diurnal Variation of Land Surface Temperature Distribution and Its Relation to Land Use/Land Cover Patterns

**DOI:** 10.3390/ijerph191912738

**Published:** 2022-10-05

**Authors:** Ruirui Dong, Michael Wurm, Hannes Taubenböck

**Affiliations:** 1 Earth Observation Center (EOC), German Aerospace Center (DLR), 82234 Oberpfaffenhofen, Germany; 2 Institute for Geography and Geology, Julius-Maximilians-Universität Würzburg, 97074 Würzburg, Germany

**Keywords:** surface urban heat island (SUHI), land use/cover pattern (LUCP), land surface temperature (LST), seasonal, diurnal

## Abstract

The surface urban heat island (SUHI) affects the quality of urban life. Because varying urban structures have varying impacts on SUHI, it is crucial to understand the impact of land use/land cover characteristics for improving the quality of life in cities and urban health. Satellite-based data on land surface temperatures (LST) and derived land use/cover pattern (LUCP) indicators provide an efficient opportunity to derive the required data at a large scale. This study explores the seasonal and diurnal variation of spatial associations from LUCP and LST employing Pearson correlation and ordinary least squares regression analysis. Specifically, Landsat-8 images were utilized to derive LSTs in four seasons, taking Berlin as a case study. The results indicate that: (1) in terms of land cover, hot spots are mainly distributed over transportation, commercial and industrial land in the daytime, while wetlands were identified as hot spots during nighttime; (2) from the land composition indicators, the normalized difference built-up index (NDBI) showed the strongest influence in summer, while the normalized difference vegetation index (NDVI) exhibited the biggest impact in winter; (3) from urban morphological parameters, the building density showed an especially significant positive association with LST and the strongest effect during daytime.

## 1. Introduction

Air and surface temperatures in urban areas are often higher than in surrounding rural areas. This phenomenon is known as the urban heat island (UHI) effect [1] which is caused due to higher shares of impervious surfaces and the intensity of usage in urban environments. Asphalt, cement, and roofing tiles, among other urban building materials, have a substantially higher heat capacity than other natural components [2]. UHIs are formed as a result of higher anthropogenic heat emissions, less evaporative cooling, increased surface roughness, lower surface albedos, and narrow urban canyon geometries as a result of urbanization. The UHIs have caused negative effects such as increased energy consumption due to air conditioning [3], air pollution [4], and water shortages as evapotranspiration increases and precipitation decreases in some desert cities. Moreover, it threatens the health of urban residents [1] and human comfortability [5,6]. For example, the continuous high temperature in summer in Arizona led to the state’s highest temperature mortality rate in the United States from 1993 to 2002, and the excessive heat event in France defined the deaths of approximately 15,000 people in the summer of 2003. While traditional approaches for evaluating urban climate relate to air urban heat islands, an increased number of studies relate to the effects of certain land cover types to describe local microclimate phenomena under the term surface urban heat island (SUHI) [7,8,9,10,11]. Especially in times of climate change and increased excessive heat events, there exists an urgent need to better understand UHIs and thus derive a larger information basis for developing mitigation strategies.

To derive area-wide data on land surface temperature (LST), earth observation satellites incorporating thermal sensors have been utilized for SUHI research (e.g., [8,9,10]). When compared to air temperatures gathered from urban weather stations, thermal imagery provides current, explicit, and area-wide data at multiple spatial and temporal scales [1,9,11], which are important prerequisites for studying SUHI. LST, which is influenced by land cover/land use type and the spatial urban structure, is one of the most obvious responses to the urban thermal environment [12,13] making these data crucial to understanding how urbanization affects the urban thermal environment. However, the mechanisms and complex interactions behind the surface temperature in urban areas are not yet fully understood.

Numerous related studies have reported that the SUHI generated by the specific urban structure, such as landscape compositions [14] and three-dimensional (3D) building morphological layout [15,16], can be related to specific land use/land cover patterns (LUCP) characteristics, making it crucial to understand how LUCP affects LST. In related studies, the normalized difference vegetation index (NDVI) and the normalized difference built-up index (NDBI) have shown strong relations with LST [6,17,18,19]. Further, the building height (BH), building density (BD), and floor area ratio (FAR) could be significantly related with LST [20,21].

Urban heat islands are not static in terms of the seasonal or even daily dynamics because the thermal response of certain surface types, such as vegetation or surface water, react with delay to changing air temperature [18,22]. The temperature differences are observed especially in summer nights [23], and it was shown that nighttime temperatures, especially, have a major effect on the UHI and the health of urban residents [24].

Previous research on the SUHI concentrated more on the phenomenon during the daytime in summer because high and moderate resolution images were widely available for this point in time. In addition, the mechanisms which drive LST are also stated to differ between day and night in cities [25,26]: various land use types are measured with varying LSTs at daytime compared to nighttime [27]. While many researchers have examined the effects of biophysical factors on the SUHI, multi-temporal examinations on season and time of day have not been systematically documented. In recent years, an increasing number of scholars have aimed at the relation of urban 3D morphology to LST [20,28]. Buildings, their physical characteristics, and their pattern modify solar radiation reflection and absorption; likewise, the roughness of the urban surface, affects surface ventilation and heat exchange. To add to the current related literature, the purpose of this study aims to statistically quantify how the LUCP indicators affect urban LST characteristics on seasonal and diurnal scales. We selected Germany’s capital, Berlin, as a case study, as it is the largest city in Germany. incorporates a broad variety of land use/cover types, and it is situated in a climate zone with high variations in daytime and nighttime temperatures as well as well high differences between summer and winter temperatures.

Given the above background, three aspects of this study are: (1) to investigate the spatial variability of LST patterns seasonally and diurnally for the city of Berlin; (2) to analyze and compare the performance of land surface temperature on various land surfaces at different times and identify its impacts on land surface temperature spatial distribution; (3) to explore quantitative associations between seasonal and diurnal LST variations and LUCP variables which determine LST intensity including land use/land cover factors and urban morphology indices; and (4) to provide valuable insight and scientific guidance for urban planners to mitigate the SUHI effects to effectively improve the thermal environment.

## 2. Data and Methods

### 2.1. Study Area

**Figure 1:**
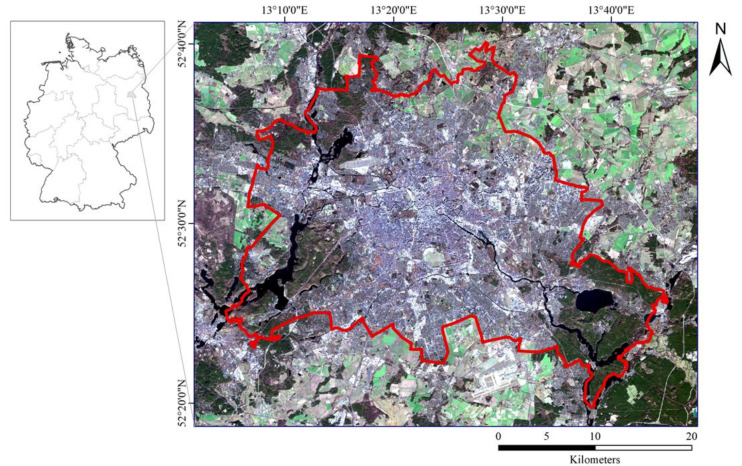
The study area of Berlin (red boundary) represented in a Landsat 8 image.

The research area is Berlin, the capital city of Germany (52.34°–52.68° N, 13.10°–13.77° E). It is located in northeastern Germany and covers an area of around 900 km^2^. With an annual mean temperature of 9.5 °C and annual precipitation of 591 mm, Berlin is deeply affected by the prevailing westerlies in summer and characterized by a transition from maritime temperate climate to the continental climates of the interior of Europe, according to the Köppen climate classification. Berlin has a population of 3.6 million residents, with one-third of them dwelling in the city center. In spite of the temperate climate, mortality rates in Berlin are up to 67.2% higher during intense heat waves [29], most notably due to increased risk of heat stress in the central city [30] because of a microclimate which is characterized by urban heat island effects. This is substantial to exhibit the diversity of land cover such as different densities of built-up areas, vegetation areas, water, etc. (Figure 1). The numerous lakes in the western and southeastern areas are the most prominent physical attributes.

### 2.2. Data Sets

This study relies on three different input data types: (1) surface temperature from thermal remote sensing, (2) land use/land cover classification, and (3) urban morphologic indicators.

**Figure 2:**
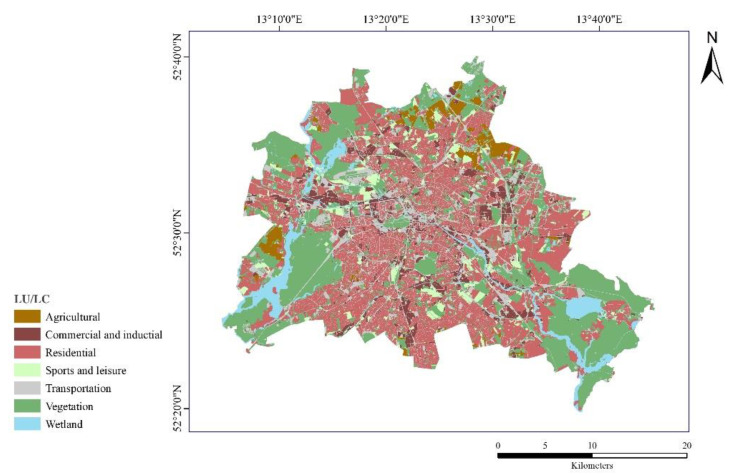
The classification of land use/land cover map in Berlin in 2018 based on the European Urban Atlas.

1. For thermal data, we rely on multi-temporal data from the Landsat 8 Thermal Infrared Sensor (TIRS) and Operational Land Imager (OLI) data, which are publicly available (level-1-products http://earthexplorer.usgs.gov/ accessed 20 May 2020). The spatial resolution of Landsat 8 image bands is 30 m except for band 8 (panchromatic, 15 m spatial resolution) and the two thermal bands 10–11 (100 m). Conditions for images to be selected for the study, are: (1) cloud-free; (2) coverage of the entire study area; (3) four-season division covering spring (March–May), summer (June–August), autumn (September–November), and winter (December–February); and (4) daytime (local overpass time before noon) and nighttime (local overpass time after 8 p.m.). Based on these criteria, the following eight images between 2018 and 2019 were selected to retrieve LSTs in spring, summer, autumn, and winter as well as day and nighttime of Berlin (Table 1). The time of data acquisition was approximately 12:00 a.m. and 10:30 p.m. Greenwich Mean Time (GMT) in Berlin.
2. The land use/land cover (LU/LC) data (10 m) of Berlin (Figure 2) in 2018 were obtained from the European Urban Atlas (https://land.copernicus.eu/local/urban-atlas accessed 18 February 2020), which provides reliable, inter-comparable, high-resolution data. The LU/LC classification includes six primary types (croplands, woodlands, grasslands, water areas, built-up lands, and unused lands) and 25 secondary types. We identified seven important classes (Table 2): Transportation (15.2%), Commercial and Industrial (6.7%), Residential (35.2%), Sports and Leisure (4.3%), Vegetation (30.2%), Agriculture (2.9%), Wetlands (5.5%) (the percentage of the corresponding class within the study area in 2018).
3. Urban morphologic indicators were obtained from building footprints from Open Street Map (https://www.openstreetmap.org/ accessed on 1 January 2020).

Berlin is characterized by high shares of impervious land and a generally low to moderate pace of urban growth between 2018 and 2019. Thus, the LU/LC changes in Berlin over these two years are fewer than the inaccuracies in the mapping results, whereas the shift in time between the LU/LC map and the building survey data can be considered negligible.

### 2.3. Research Framework

**Figure 3:**
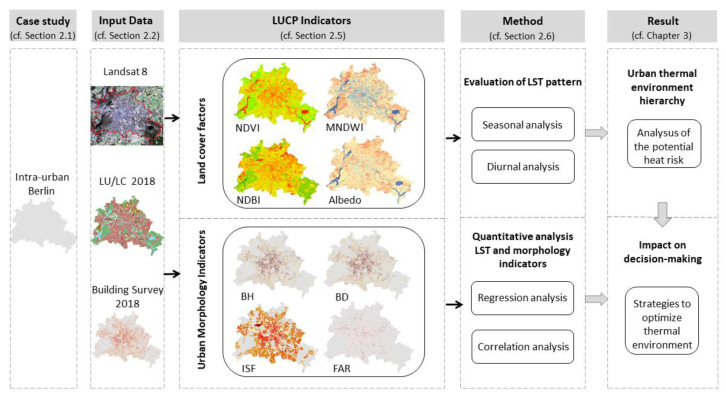
The conceptual framework to investigate statistical relationships between LUCP indices and LST characteristics.

The following conceptual framework, as shown in Figure 3, provides an overview on the three essential analysis steps:

(1) Evaluating of the pattern of the urban thermal environment by retrieving the LST from Landsat images and analyzing the corresponding spatial patterns for multiple points in time at the inner-city scale;

(2) Obtaining 2D metrics (i.e., NDVI, NDBI, and albedo) as well as 3D indicators (i.e., height, volume, and density), respectively, from Landsat images, land use/land cover data, and building survey data; and

(3) Conducting statistical analysis (correlation and regression) to estimate the seasonal and diurnal associations between LST and these indicators.

### 2.4. Retrieving Land Surface Temperature

Considering that LST is sensitive to the atmospheric effects, and in order to obtain accurate and consistent spectral information [31], atmospheric correction is necessary to convert the top-of-atmosphere reflectance to surface reflectance. The FLAASH (Fast Line-of-Sight Atmospheric Analysis of Spectral Hypercubes) model provided by the ENVI software was used for the atmospheric correction, which is based on MODTRAN (Moderate Spectral Resolution Atmospheric Transmittance Algorithm and Computer Model). The computation of at-sensor spectral radiance is required to convert image data from multiple sensors and platforms into a physically appropriate common radiometric scale. The following equations were used to convert the digital numbers for both reflective and thermal bands to top-of-atmosphere radiance [32]:

(1) $$L_{\lambda }=M_{L}Q_{Cal}+A_{L}$$

where $L_{\lambda }$ is top-of-atmosphere radiance at the sensor’s aperture in W/(m^2^·sr·μm), $M_{L}$ is the band-specific multiplicative rescaling factor from the satellite metadata equal to 0.0003342, $Q_{Cal}$ is the pixel digital number for thermal band 10, $A_{L}$ is band-specific additive rescaling factor from the satellite metadata.

The following equation is used to convert the spectral radiance $L_{\lambda }$ to the at-sensor brightness temperature [32]:

(2) $$T_{BT}=\frac{K_{2}}{\ln\left(\frac{K_{1}}{L_{µ}}+1\right)}\;$$

where, $T_{BT}$ is the effective at-sensor brightness temperature in Kelvin, $L_{µ}$ is the spectrum radiance at the sensor’s aperture in W/(m^2^·sr·µm), and $K_{1}$ and $K_{2}$ are the pre-launch calibration constants. For Landsat 8 OLI, $K_{1}$ = 774.89 W/(m^2^·sr·µm) and $K_{2}$ = 1321.08 K.

The temperature values were derived using a black body, which has properties that are different from that of real objects. The land surface temperature was calculated after correction for spectral emissivity (ε) of a grey body was implemented [33,34]:

(3) $$T_{LS}=\frac{T_{BT}}{1+\left(\lambda *\frac{T_{BT}}{\alpha }\right)\ln\varepsilon }-273.15$$

where $T_{LS}$ is the land surface temperature, $T_{BT}$ is the black body temperature in Kelvin, λ is the wavelength of radiance emitted (which for Landsat 8, band 10 is 10.8, and band 11 is 12), α = hc/b (1.438 × 10^−2^ mK), h = the Planck’s constant (6.626 × 10^−34^ J/s), c = velocity of light (2.998 × 10^8^ m/s), b = Boltzmann constant (1.38 × 10^−23^ J/K), and ε = surface emissivity.

Land surface emissivity is a key parameter in the measurement of LST. An accurate estimate of surface emissivity is crucial for the reliable derivation of the surface temperature. Water, vegetation, and roughness are a few aspects of the variables that affect a surface’s emissivity [35]. Empirical approaches [36,37] were used to discover a relationship between emissivity and the value of NDVI in the scientific literature. This emissivity estimating method was utilized in TM/ETM+ band 6, however, because of their approximate spectral range, it can also be applied to Landsat 8 band 10 [38]:

(4) $$\varepsilon =\left\{\begin{array}{c} \;\;\;\;\;\;\;\;\;\;\;0.995,\;\;\;NDVI<0\;\;\;\\ \;0.9589+0.086*P_{v}-0.0671*P_{v}{}^{2},\;\;\;0\leq NDVI\leq 0.7\\ 0.9625+0.0614*P_{v}-0.0461*P_{v}{}^{2},\;\;\;NDVI>0.7 \end{array}\right.$$

where ε is land surface emissivity and $P_{v}$ is, according to Sobrino, Jimenez-Munoz, and Paolini [39], the vegetation proportion obtained:

(5) $$P_{v}={\left(\frac{NDVI-NDVI_{min}}{NDVI_{max}-NDVI_{min}}\right)}^{2}$$

where $NDVI_{max}$ and $NDVI_{min}$ are the maximum and minimum vegetation index in the study area, respectively.

### 2.5. Selected LUCP Indicators

Different land cover types reflect various heat capacities and evapotranspiration ratios. The roughness of the urban surface and urban air ventilation conditions are reflected in several morphological indices. Nonetheless, each of these types of indicators have different effects on urban LST, which together moderate the urban surface thermal climate [16].

For vegetation, we apply the commonly used NDVI [40], which relates to the density of vegetation. For the built landscape, we apply the NDBI [41] which is a measure for the intensity of the built-up area. For water, we apply the modified normalized difference water index (MNDWI), which represents the amount of water state of vegetation [42,43,44]. MNDWI has been identified to be preferable compared to the normalized difference water index (NDWI) for characterization of the original biophysical water index since the latter tends to extract water cover combined with built-up area characteristics [19]. Further, albedo is an essential property of the land surface heat budget [45].

Beyond the ratios from land cover compositions introduced above, we apply parameters further describing spatial morphology of the urban environment. To better define the urban morphological factors of LST from building survey data, we calculate the following indicators: Fraction of impervious surface (ISF), which quantifies the fraction of impervious surface in percent; BD, which quantifies the total area of building ground floors per reference unit in percent; and FAR, which quantifies the total area of building floors per reference unit [46]. All factors selected in this study are presented in Table 3.

### 2.6. Statistical Analyses Relating Spatial Indicators and LST

In this study, two common statistical approaches were used to quantify associations between LST and selected indicators on multi-temporal scales. For this, the optimal pixel size for simulating environmental characteristics between LST and LUCP indicators must be considered. The ideal geographical scale for examining LST and ISF relationship was determined to be 500 m, as proposed by relevant research [14], while the optimal size of the green space that effects LST effectively was found to be 210 to 240 m [48]. Generally, at coarser scales a stronger correlation has been already shown [49]. Thus, in this study we used 240 m as the window size for LST and the land cover factors, while for LST and the urban morphologic indicators we applied 500 m. As advised by related studies [8,50], the pixel aggregation tool provided by ENVI was used to perform the resampling. The tool uses weighted average to aggregate the input cell values that contribute to the output grid values, removing the effect of nearby pixels.

Pearson correlation, a frequently employed statistical method, was utilized to evaluate the relationship between each selected factor and LST. Pearson’s correlation coefficient is a measurement of the linear correlation between two variables [51]. The Pearson correlation coefficient is calculated as follows [52]:

(6) $$r=\frac{\sum \left(x-\bar{x}\right)\left(y-\bar{y}\right)}{\sqrt{\sum {\left(x-\bar{x}\right)}^{2}\sum {\left(y-\bar{y}\right)}^{2}}}\;$$

where *r* is the correlation coefficient, *x* and *y* represent the selected factors and LST, and $\bar{x}$ and $\bar{y}$ are the mean values of *x* and *y*, respectively.

Ordinary least squares (OLS) regression model has been employed to examine how land cover factors affect the LST. The corresponding slope coefficients produced from the models are used to assess the localized contribution of each indicator to LST. Improved OLS regression models [53,54] use the equation:

(7) $$Y=\beta_{0}+\underset{n}{\sum }\beta X_{n}+\varepsilon \;$$

where *Y* denotes LST, $\beta_{0}$ is the intercept value, β represents slope coefficient, which means how the LST changes linearly with each indicator, $X_{n}$ represents each selected indicator, *n* represents the number of indicators, and *ε* is the random error term.

A multi-scale geo-weighted regression model (MGWR) is used to describe the non-stationary characteristics of spatial data [55], which enables us to explore the relationship between building form layout parameters and LSTs. The MGWR models were built on the open-source platform GWR 4 (https://sgsup.asu.edu/sparc/gwr4 accessed on 2 March 2022), which is expressed as:

(8) $$Y_{i}=\beta_{0}\;\left(u_{i}+v_{i}\right)+\overset{n}{\underset{k=1}{\sum }}\beta_{bwk\;}\left(u_{i}+v_{i}\right)\;x_{ik}+\varepsilon_{i}\;$$

where $\left(u_{i}+v_{i}\right)$ denotes the coordinates of the *i*-th point in space, $\beta_{0}\;\left(u_{i}+v_{i}\right)$ is the intercept value, $\beta_{bwk\;}$ represents the ideal bandwidth for the association modeling between the *k-*th indicator and LST, $x_{ik}$ is the *k-*th indicator at observation *i*, $\varepsilon_{i}$ is the random error term at point *i*, and *n* is the number of independent variables.

## 3. Results

### 3.1. Spatial Distribution of LST

We estimated the mean LST for daytime and nighttime in all seasons, respectively, to investigate seasonal and diurnal aspects of LST variations. The maximum, minimum, and average temperatures in Berlin’s LST differ significantly among the four seasons. Not surprisingly, the LST of summer was the highest, followed by the transition seasons (spring and autumn), and then winter. The standard deviation (Std) of LST, observed in summer and in the transition seasons are higher than in winter during daytime (Table 4). However, the standard deviations observed in summer and autumn are lower than in spring and winter at nighttime. 

Although seasonal changes might affect absolute LST measurements, it is difficult to compare the magnitude and variation of LST over seasons intuitively using absolute LST. The seasonal difference, however, has no impact on the distributional pattern of LST. The standard deviation and mean value can be used to illustrate how various LSTs vary. The thermal landscape was classified into six levels using the mean-Std criterion [56]:

(1) Very Hot Spot: LST ≥ LST_mean_ + 2Std;
(2) Hot Spot: LST_mean_ + Std ≤ LST ≤ LST_mean_ + 2Std;
(3) Warm spot: LST_mean_ ≤ LST ≤ LST_mean_ + Std;
(4) Cool Spot: LST_mean_ − Std ≤ LST ≤ LST_mean_;
(5) Cold Spot: LST_mean_ − 2 Std ≤ LST ≤ LST_mean_ − Std;
(6) Very Cold Spot: LST ≤ LST_mean_ − 2Std.

**Figure 4:**
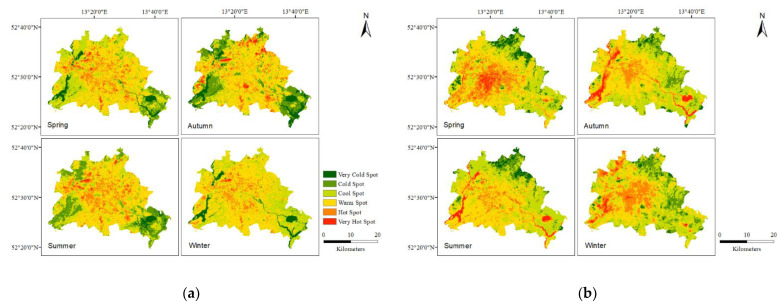
The spatial distribution of LST in (**a**) daytime and (**b**) nighttime.

According to this classification scheme, the seasonal and diurnal LSTs generated from Landsat thermal bands in Berlin are mapped and illustrated in Figure 4. It is known that LST and LULC are related [57] and we can observe this by the highest measured LSTs which are found mostly in areas with high amounts of impervious land such as commercial or industrial areas. Further, hot spots were identified around the high impervious surface fractions at Tegel Airport and at commercial and industrial centers in the west and south.

Warm spots have the largest spatial extent in summer and the transition seasons, and cool spots have the largest spatial extent in winter. During the nighttime, the LST value gradient from the countryside to the central commercial areas is measured. The coldest spots are located in forest zones and open leisure spaces such as in Köpenick district. The cool zones are concentrated in districts with lower density of built-up areas. Dense residential and commercial areas in the downtown area contributed to warm spots. All of the highest LST values were clustered in water body areas. In summary, hot spots occurred at the built-up locations where, with high impervious surface fractions and dense population, this impact is induced by increased solar radiation absorption, increased infrared radiation retention, and delayed heat release [58]. Additionally, thermal inertia in water bodies is higher at night, slowing heat transmission.

### 3.2. Land Cover Analysis of LST

**Figure 5:**
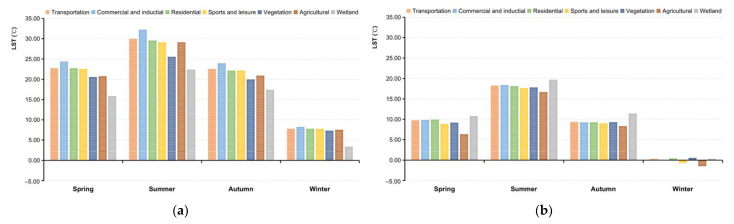
Seasonal variability of the average LSTs by different land cover type in (**a**) daytime and (**b**) nighttime.

The complex interaction of urban morphology with the surrounding environment [59], as well as the distinctive LST responsible for the thermal properties of land cover types, contribute to the urban climate. The seasonal and diurnal LSTs of each land cover type were analyzed in Figure 5.

The impervious surface areas, mainly including transportation, industrial, commercial, and residential areas, produced the highest daytime LST across four seasons, followed by agricultural land (Figure 5a). The lowest LSTs at daytime were constituted in wetlands, followed by vegetation including meadows, gardens, and forests. Vegetation in summer was shown to have the strongest cooling effect, followed by spring and autumn. These findings imply that an increase in LST is caused by intensive impervious surfaces associated with human activity. Vegetation and water bodies were the main sources of cooling during the daytime by decreasing latent heat fluxes [60]. The highest temperatures are detected in locations surrounding water bodies, according to nighttime LST maps (Figure 5b). Thermal inertia in water bodies is highest at night, slowing heat transfer by contrast in the daytime. Agricultural areas are very cold spots with the lowest temperature. The LST of agriculture was lower than vegetation at night but higher in the daytime. Except for water bodies, the LST difference of other surface types at night is much smaller than that during the daytime.

**Figure 6:**
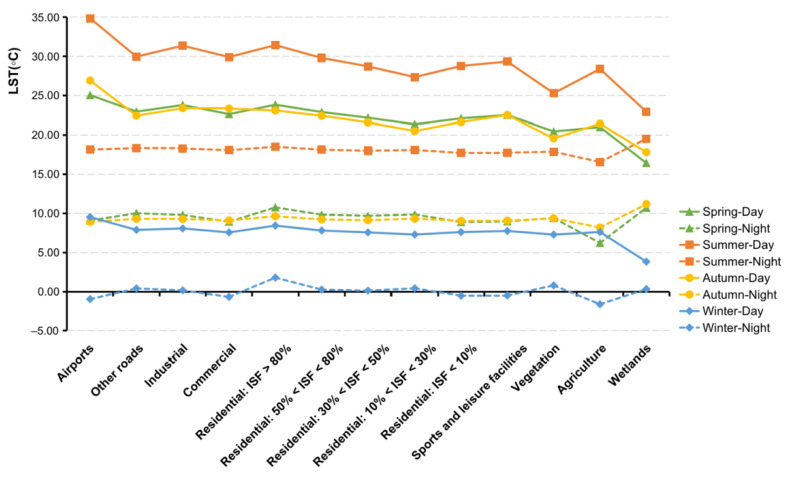
Seasonal variability of the average LSTs by detailed land cover type.

LSTs fluctuated for different land cover types at different timescales in different manners. Figure 6 demonstrates the LST in a more detailed way for the various land cover types for all seasons at nighttime as well as daytime. Comparing the curvature of the folds reveals that the seasonal effects of LST are larger than effects caused by the land cover type, especially at nighttime. The central business district, industrial lands, traffic hubs, and high-density residential area are observed to correspond to higher LST values. Higher LST result in wider temperature gradients: These exacerbate sensible heat fluxes in the absence of water sources for evapotranspiration. This emphasizes the significant influence of human activities on the urban thermal environment. Compared to the nighttime, the LST measured on impervious surface areas in the daytime displayed a noticeable reduction as the impervious surface fraction decreased. Specifically, the diurnal temperature range was smaller in low-intensity residential areas, apparently in summer. In addition, it was found that the variance between day and night in the water bodies is the smallest in any season. Since light can access deep into water without remarkable heat flow during the daytime, it results in colder surface temperatures and thus, water bodies and wetlands are recorded with the lowest temperature. In other words, water bodies have the highest specific thermal capacity as well as the slowest cooling rate [61]. To maintain thermal balance, it collects solar radiation during the daytime and releases heat at night, resulting in higher LSTs across the research area. During the nighttime, water bodies show a heat effect and they have a cooling effect during the day. This can be explained by the diurnal LST differences causing variances in thermal inertia [62].

### 3.3. Spatial-Temporal Patterns of LUCP Indicators

**Figure 7:**
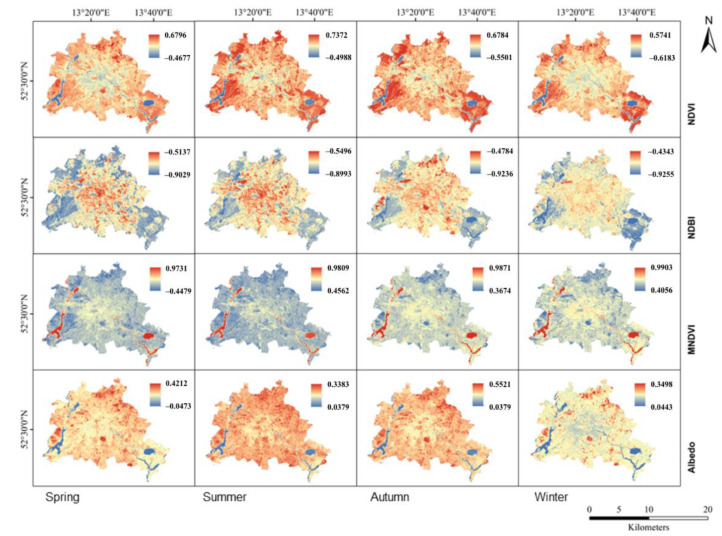
The spatial distributions of the land cover factors reflect distinct seasonal variations in daytime.

**Figure 8:**
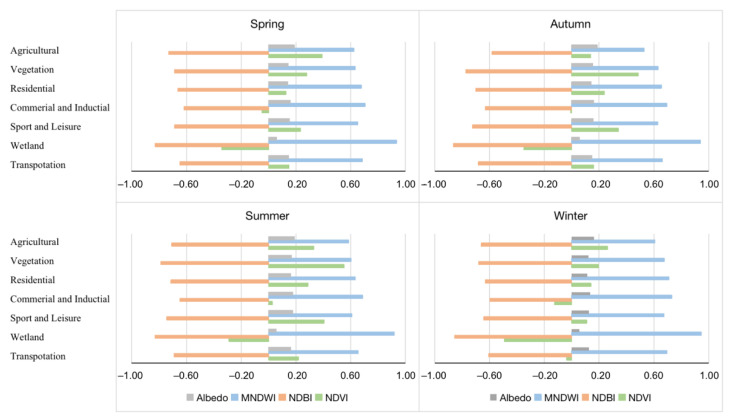
The season variation of the land cover factors by land cover type in daytime.

The LU/LC heterogeneity of the research area is illustrated using NDVI, NDBI, MNDWI, Albedo, ISF, BH, BD, and FAR. NDVI, NDBI, MNDWI, and Albedo, clear seasonal fluctuations are mapped in Figure 7 and plotted by land cover type in daytime in Figure 8. 

Naturally, the spatial patterns of NDVI varied over the seasons. While in spring and autumn the NDVI was measured at similar values, the NDVI is recorded with a seasonal cycle related to biological activity. The NDVI value varies from −1 to 1. Higher NDVI values mean higher dense greenery. NDVI values show an increasing gradient from the center to the periphery; however, local variations due to the airport or commercial centers exist (Figure 7). In our measurements, we found that when NDVI values increased due to a higher share of vegetated areas, LST values decreased; vice versa, higher values of NDBI related to denser built-up areas, coincided with higher LST values. For the seasonal variations of MNDWI, we found green vegetation and water bodies generally opposite to the NDVI, but the spatial distributions of the impervious surfaces were roughly the same in the four seasons. The spatial distribution of MNDWI showed much greater variability in summer than in other seasons, especially in the central city. Although there was a slight seasonal variation in water temperature during the transitional and winter seasons, water bodies remained rather steady. Low albedo values were associated with wetlands as well as developed areas with higher albedo, according to the statistics. In particular, summer was the time when the highest albedo areas were observed.

**Figure 9:**
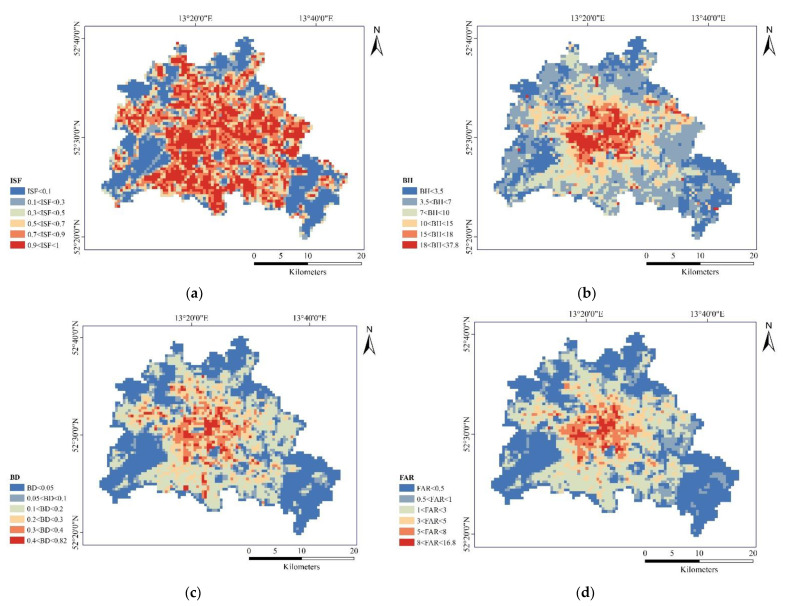
The spatial distributions of urban morphology indicators: (**a**) ISF; (**b**) BH; (**c**) BD; (**d**) FAR.

We illustrate the heterogeneity of the built environment by the variables ISF, BH, BD and FAR (Figure 9). In general, the spatial distribution of BD was most consistent with the accompanying LST pattern.

### 3.4. Correlation between LST and LUCP Indicators

We further analyzed the relationship between eight impact factors on the distribution of Berlin’s LST by correlation and regression analysis at the pixel level. Surface heat storage is closely related to the heat capacity and thermal properties in both natural landscapes and layout of buildings [63]. In all models, NDVI, NDBI, MNDWI, and albedo were all significant at the 0.01 confidence level.

**Figure 10:**
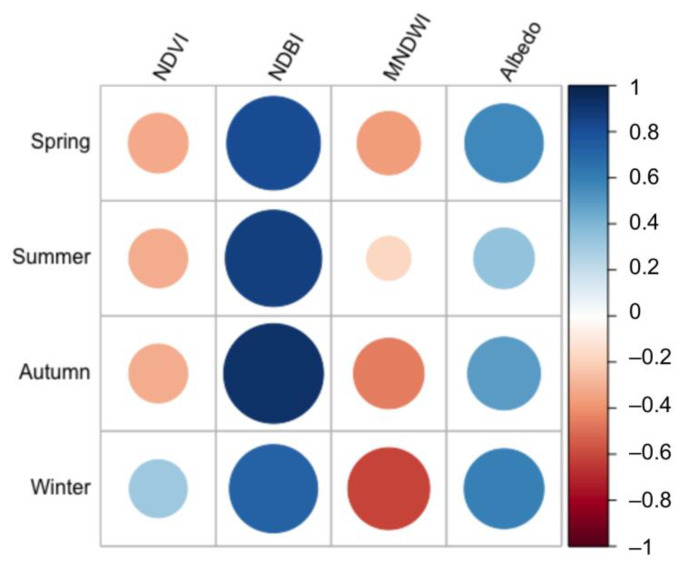
Plot of the correlation coefficient of four land cover indices in daytime.

Table 5 and Figure 10 reveal negative correlations of LST with NDVI and MNDWI, which are in accordance with what earlier research has revealed [64]. In the correlation between LST and NDVI, it can be clearly seen that there is a positive correlation in winter and a negative correlation in other seasons during the daytime. Since crops are harvested in autumn, the considerable decline in vegetation coverage comes with a decreased ability to cool the surface [65]. A negative association was identified between LSTs and MNDWI in all seasons. In the correlation between LST and MNDWI, it was obvious that there had been an increase in the absolute value of the correlation coefficient from summer to winter, with the lowest in summer, followed by spring and autumn, and the highest in winter. In contrast, a positive correlation existed between NDBI and LST. The regression coefficients (R^2^) in four seasons were 0.97, 0.97, 0.96 and 0.93, respectively, and it was indicative of the strong association between LST and NDBI. Albedo also showed a positive effect in all seasons, and the association in spring and winter tended to be more positive than in summer. Lower albedo leads to hotter values and this was demonstrated at the building level [66].

**Figure 11:**
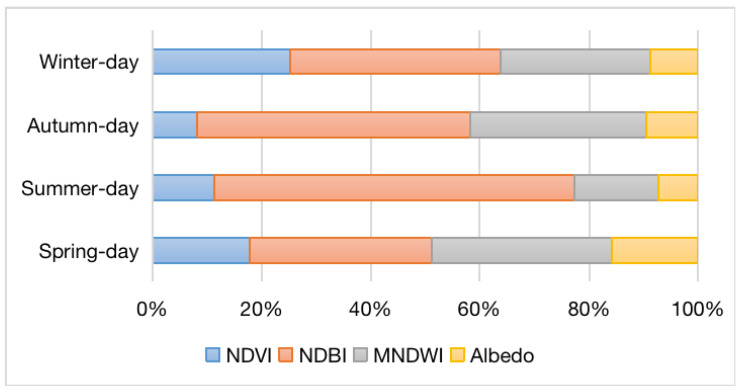
Comparison of the contributions of land cover indices regarding the LST by standardized regression coefficients.

The contribution of each impact factor on LST change was measured using the standardized regression coefficients. Their impact on the four seasons was not entirely constant, as seen in Figure 11.

In spring, NDBI (0.335) > MNDWI (0.330) > NDVI (0.177) > Albedo (0.158);
In summer, NDBI (0.660) > MNDWI (0.155) > NDVI (0.112) > Albedo (0.073);
In autumn, NDBI (0.502) > MNDWI (0.321) > NDVI (0.082) > Albedo (0.095);
In winter, NDBI (0.388) > MNDWI (0.274) > NDVI (0.251) > Albedo (0.087).

Generally, NDBI has the highest impact on LST, which supports the fact that high LST cannot be detached from impervious surfaces. This is followed by MNDWI and NDVI. To some extent, this demonstrates the relevance of the water bodies and vegetation in regulating urban temperature. In summary, NDBI shows the greatest influence in summer, while NDVI exhibits the biggest impact in winter.

In general, we found positive correlations between ISF, BH, BD, and FAR and LST whether in daytime or nighttime, which gradually strengthened with increased temperature (Table 6). All models are significant at the 0.01 confidence level.

The correlation model of ISF is the most significant between urban morphology indicators and LST, which proves that, independent of season or time of day, higher ISF values increase LSTs [17,49,61,67]. Generally, the heating effect of impervious surfaces in spring and summer were stronger than in autumn and winter. During the daytime, the heat fluxes are predominantly generated by locations having a higher ISF, which are more able to absorb solar energy. This is owing to the lack of water on these surfaces for latent heat loss, as well as the fact that a greater ISF indicates a lower vegetation proportion within the grid. The majority of the heat is provided at night by the energy accumulated all day and human activity such as transportation and manufacturing, both of which are primarily connected to ISF. BD also showed a remarkable positive association model in this study, which is consistent with ISF in seasonal and diurnal variation. BH demonstrated the lowest correlation with LST among all urban morphology indicators in the daytime. It is worth noticing that the effect of BH increased LST in spring daytime and winter nighttime. FAR was positively associated with LST in all seasons, especially in summer daytime and winter nighttime.

**Figure 12:**
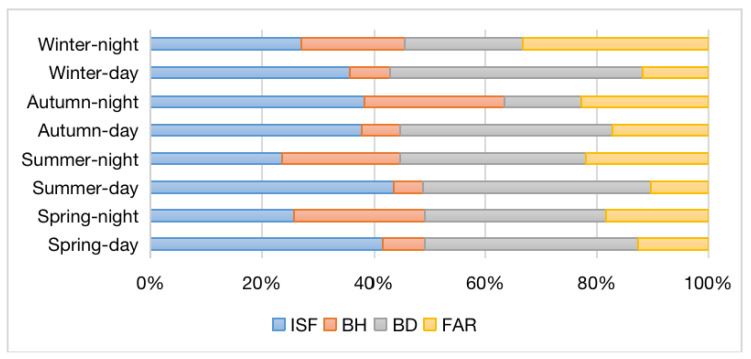
Comparison of the contributions of urban morphology indicators regarding the LST by standardized regression coefficients.

The standardized spatial non-stationary slope coefficients denoted the contributions of the urban morphology indicators to LST, as is shown in Figure 12. Generally, in the daytime, ISF > BD > FAR > BH, but in winter, BD (0.454) > ISF (0.357) > FAR (0.118) > BH (0.071). On the contrary, this observation did not exist in the nighttime: BD (0.324) > ISF (0.258) > BH (0.234) > FAR (0.184) in spring;

In summer, BD (0.334) > ISF (0.235) > FAR (0.220) > BH (0.211);
In autumn, ISF (0.384) > BH (0.249) > FAR (0.227) > BD (0.139);
In winter, FAR (0.333) > ISF (0.271) > BD (0.212) > BH (0.183).

Generally, among all analyses, BD has the strongest effect on LST during the daytime, followed by ISF, which displayed the strong impact at night, whereas BH exhibits the smallest impact on LST. FAR shows the most impact on LST in winter daytime in particular.

## 4. Discussion

This research focuses on the spatial relations between LUCP indicators and LST during daytime and nighttime and among various seasons in order to describe the urban thermal environment variations using the example of Berlin.

### 4.1. Investigation of Seasonal Variations in Urban Thermal Environment

This research investigates diurnal and seasonal LST variations, as well as associations between LST and four physical factors and four urban morphology indicators over the four seasons in Berlin. The study verified the contributions of diverse landscapes to the urban thermal environment and thus can provide additional data for urban planning. The correlation analyses between land cover indices indicate that impervious surfaces, water bodies, and vegetation play the most vital role in determining the LST dynamic. The ISF and BD are the most significant in explaining variables among selected urban morphology indices. The slope coefficients between ISF and LST and between BD and LST were around 0.235–0.435 and 0.139–0.409, respectively.

In general, hot spots of LSTs developed almost consistently with the locations of urbanized areas (Figure 4). Impervious surface was the most significant source of heat in the urban thermal environment. Beyond that, it was shown that it was dramatically higher in the summer daytime than in other seasons. Solar radiation is the primary source of heat during the daytime. However, the nighttime SUHI was related to heat accumulation during the daytime and anthropogenic heat emission from the metropolis [60]. LST, for example, always shows positive correlations between ISF and BD. As opposed to rural regions, downtown areas have higher LSTs and lower Albedo. Vegetated spaces, in comparison to constructed land, including main roads and buildings, increase latent heat flux through transpiration, resulting in cooling consequences on the LST, mitigating the SUHI effect [68,69]. Another point is shadows of canopies, which reduce the temperature [70]. Because of thermal inertia, the diurnal LST differences in water bodies were proven to have a major cooling effect during the daytime and a heating component at night. Thermal inertia in water bodies is higher at night, slowing heat transmission, and delaying heat release. Previous studies [19,71] have demonstrated that physical features such as water bodies and vegetation play a key role in alleviating SUHI. This was also demonstrated in this study, where negative correlations between LST and NDVI and MNDWI were most pronounced in summer.

### 4.2. Implications for Urban Planning and Management

In the context of climate change and the likely increase in the occurrence of climate extremes, as well as the aging of the urban population, SUHI effects represent a serious deterioration of the quality of life for many cities [72,73]. Adaptation and mitigation strategies are required, particularly in high-risk areas. The main cause of SUHI in metropolitan areas is anthropogenic heat emission [74]. While vegetation and water bodies are crucial determinants of LST during the daytime, impervious surfaces accounted for the most spatiotemporal diversity in LST at night. Surfaces with limited vegetation, along with higher ISF, are the primary producers of urban heat. As has been proven by this study, these insights can lead to a number of effective measures for reducing the urban heat island and its adverse consequences.

Firstly, removing impervious surfaces such as pavement cannot be considered a solution while keeping cities functional. However, strategies to integrate more vegetation in the urban context in appropriate locations need to be developed. According to previous studies [75,76], this is the most extensive approach used for alleviating SUHI. Further, the results suggest that increasing the ratio of high pervious surfaces, such as parking lots, with pervious surfaces, can alleviate the heat accumulated on various surfaces during the daytime. Cool and green roofs, can also be effective SUHI mitigation strategies, as well as contributing to enhancing the thermal comfort of non-cooled structures [77]. Thirdly, water bodies have also been found to be a valuable mechanism for minimizing urban heat. The integration of open water bodies close to impervious surfaces like construction supports decreasing the use of energy to keep cooling in the summer. Finally, specific adaptation and mitigation techniques are required for areas of hot spots in the summer. Changing the local environmental context, such as improving the vegetation density with street trees, roof or wall greenery [78], or reducing the vulnerable groups who dwell in this neighborhood, might be strategies.

### 4.3. Limitations and Future Studies

In spatial sciences, the quality of input data and their characteristics in terms of thematic and spatial resolution naturally influence the results [79]. For the different seasons in this research, a single impervious surface fraction classification was applied. However, since spectral characteristics are insensitive to seasonal changes, impervious surfaces were referred to as pseudo-invariant parameters in a previous study [80]. However, it has been reported that the ISF in the early spring and summer TM images differed [61]. As a result, more research is in demand to examine the variability in ISF over all seasons.

Beyond these data related limitations, the following aspects are of relevance. On the one hand, this study has been carried out in one large city. However, as the factors affecting the heat island effect of major cities may be more complicated, involving not only climate and urban morphology, but also related mitigation strategies, the findings should be systemized for more large cities around the world. Comparative studies among different cities will allow confirmation of these results for different climatic conditions or different structural patterns. However, the connection between the architectural landscape and the local LST impact mechanism in overheating areas needs to be investigated in more detail. In addition, population density and socioeconomic variables need to be considered in order to improve and propose specialized urban planning strategies. Future urban planners and city inhabitants will be constantly challenged by demographic and climatic change, demanding innovative adaptation and mitigation measures [81].

## 5. Conclusions

This study assessed the seasonal and diurnal variations of the severity of the SUHI in relation to the urban land use/cover and the urban morphology by employing the indicators for the sample city of Berlin. Based on statistical analysis, associations between LST and four land use/land cover factors and four urban morphology indicators were investigated. Four conclusions were drawn from this study:

Firstly, the LSTs in Berlin showed clear seasonal and diurnal differences in spatial distribution, with thermal hot spots primarily in districts of high shares of impervious surfaces during the daytime and, in contrast, in water bodies during nighttime. Secondly, within areas of high imperviousness, the diurnal temperature range in commercial and residential areas were lower than in transportation and industrial areas, and, naturally, higher than in vegetation areas, the water body was measured with the lowest range in any season. The temperature differences across the seven investigated land cover types were much higher at daytime than at nighttime. Thirdly, among the investigated land cover indices, NDBI, MNDWI, and NDVI related to built-up areas, water bodies, and greenery. These were the key variables determining different distributions of temperature. From the positive or negative correlation, water areas and green spaces play an extraordinary role in alleviating urban thermal environment. Finally, from the perspective of urban morphology, four indicators were found to explicitly have a heating effect on LST during daytime as well as during nighttime: ISF and BD revealed the most evident positive association. BH was found to have the least influence on LST among all indicators.

Based on the empirical results highlighted above, the study reveals how different indicators impact the urban thermal environment at different seasons and at different times of the day and night. The local distinction of LST mechanism for different periods may provide urban planners in Berlin with useful information and, in general, effective reference for implementing specific mitigation measures.

## Figures and Tables

**Table 1:** Data source information on remote sensing images.

Satellite Sensor	Date	Path/Row	Season	Time
Landsat 8 OLI/TIRS	2018/04/18	193/23	Spring	Day
	2018/09/09	193/23	Autumn	Day
	2019/02/15	49/221	Winter	Night
	2019/02/16	193/23	Winter	Day
	2019/04/20	49/221	Spring	Night
	2019/06/23	49/221	Summer	Night
	2019/06/24	193/23	Summer	Day
	2019/09/27	49/221	Autumn	Night

**Table 2:** Classification of urban land use types.

Land Cover Type	Description
Transportation	Any type of traffic land, including main roads, highways and airport
Commercial and Industrial	Urban built-up areas, including commercial land and industrial land
Residential	Urban built-up areas, including all types of residential land
Sports and Leisure	Any type of vegetation that provides pervious surface and public service land
Vegetation	Any type of vegetation that provides shade, including all trees and shrubs
Agriculture	All agricultural land
Wetlands	Any type of water body, including lakes, rivers, wetlands, and ponds

**Table 3:** Detailed description about the selected indicators.

	Indicators	Description	Value
Land cover factors	NDVI	Measures density of green vegetation, calculated as [40] $NDVI=\frac{\rho \left(NIR\right)-\rho \left(RED\right)}{\rho \left(NIR\right)+\rho \left(RED\right)}$	[−1, 1]
	NDBI	Measures intensity of imperviousness, calculated as [41] $NDBI=\frac{\rho \left(SWIR1\right)-\rho \left(NIR\right)}{\rho \left(SWIR1\right)+\rho \left(NIR\right)}$	[−1, 1]
	MNDWI	Measures characterize the water body features, calculated as [47] $MNDWI=\frac{\rho \left(GREEN\right)-\rho \left(SWIR\right)}{\rho \left(GREEN\right)+\rho \left(SWIR\right)}$	[−1, 1]
	Albedo	Overall reflectance in all directions [45] $\begin{array}{cc} Albedo=0.356 & *\rho \left(BLUE\right)+0.130*\rho \left(RED\right)+0.373\\ & *\rho \left(NIR\right)+0.085*\rho \left(SWIR1\right)+0.072\\ & *\rho \left(SWIR2\right)-0.0018 \end{array}$	[0, 1]
Spatial morphological factors	ISF	Fraction of impervious surface in each grid	[0, 1]
	BH	Average building height in each grid	[0, Max]
	BD	The building square footage divided by total land area	[0, 1]
	FAR	The building floor area within each grid	[0, Max]

**Table 4:** The statistical overview of the collected LSTs for all seasons (Date format: YYYY/MM/DD).

Season	Daytime-Date	Maximum (°C)	Minimum (°C)	Mean (°C)	Standard Deviation (°C)
Spring	2018/04/18	37.44	7.05	21.82	2.23
Summer	2019/06/24	44.89	14.51	28.26	3.34
Autumn	2018/09/09	34.62	8.04	21.57	2.39
Winter	2019/02/16	15.83	−10.90	7.47	1.34
**Season**	**Nighttime-Date**	**Maximum (°C)**	**Minimum (°C)**	**Mean (°C)**	**Standard Deviation (°C)**
Spring	2019/04/20	13.59	−9.24	9.51	1.48
Summer	2019/06/23	21.29	5.53	18.08	0.81
Autumn	2019/09/27	12.58	−0.31	9.33	0.81
Winter	2019/02/15	6.38	−17.78	0.26	1.42

**Table 5:** The relationship between the selected land cover indices and LST by Pearson correlation coefficient (white lines) and OSL coefficient of determination (gray lines).

Season	Daytime-Date	NDVI	NDBI	MNDWI	Albedo
Spring	2018/04/18	−0.33	0.81	−0.37	0.57
		0.65	0.97	0.97	0.98
Summer	2019/06/24	−0.32	0.86	−0.18	0.34
		0.63	0.97	0.97	0.97
Autumn	2018/09/09	−0.32	0.92	−0.46	0.49
		0.54	0.96	0.96	0.98
Winter	2019/02/16	0.31	0.72	−0.62	0.59
		0.06	0.93	0.92	0.96

**Table 6:** The relationship between the selected morphology factors and LST by Pearson correlation coefficient (white lines) and local R^2^ values of the MGWR (gray lines).

Season	Daytime-Date	ISF	BH	BD	FAR	Nighttime-Date	ISF	BH	BD	FAR
Spring	20180418	0.71	0.39	0.69	0.56	20190420	0.39	0.51	0.46	0.49
		0.78	0.80	0.66	0.48		0.77	0.83	0.66	0.50
Summer	20190624	0.69	0.43	0.69	0.59	20190623	0.31	0.38	0.35	0.38
		0.79	0.81	0.67	0.49		0.74	0.79	0.62	0.45
Autumn	20180909	0.43	0.23	0.47	0.37	20190927	0.23	0.30	0.17	0.24
		0.76	0.79	0.65	0.47		0.73	0.80	0.62	0.46
Winter	20190216	0.44	0.33	0.50	0.43	20190215	0.16	0.56	0.43	0.54
		0.77	0.81	0.66	0.49		0.13	0.32	0.28	0.38

## Data Availability

Not applicable.
